# Achieving High Performance of ZnSnO Thin-Film Transistor via Homojunction Strategy

**DOI:** 10.3390/mi14122144

**Published:** 2023-11-23

**Authors:** Wengao Pan, Guoshang Zhang, Xinhua Liu, Kexing Song, Laiyuan Ning, Shuaifang Li, Lijia Chen, Xuefeng Zhang, Tengyan Huang, Huan Yang, Xiaoliang Zhou, Shengdong Zhang, Lei Lu

**Affiliations:** 1 Henan Key Laboratory of Advanced Conductor Materials, Institute of Materials, Henan Academy of Sciences, Zhengzhou 450046, China; wgao_pan@126.com (W.P.); 9902991@haust.edu.cn (G.Z.); liuxinhua18@163.com (X.L.); kxsong@hnas.ac.cn (K.S.); 2 School of Electronic and Computer Engineering, Peking University, Shenzhen 518055, China; huangty@stu.pku.edu.cn (T.H.); yang_huan@pku.edu.cn (H.Y.); zhouxiaoliang@pku.edu.cn (X.Z.); zhangsd@pku.edu.cn (S.Z.); 3 School of Materials Science and Engineering, Zhengzhou University, Zhengzhou 450001, China; ninglaiyuan@lysifon.com (L.N.); lishuaifang@fonlinkcn.com (S.L.); ljchen@dgut.edu.cn (L.C.)

**Keywords:** zinc-tin oxide, oxygen vacancy, homojunction, thin-film transistor

## Abstract

The zinc-tin-oxide (ZTO) thin-film transistor (TFT) is one of the most promising candidates for advanced display applications, though its popularity is limited by its performances. In this work, a heterojunction channel strategy was adopted to regulate the electron transport behaviors and the TFT performances by manipulating the concentration and the distribution of oxygen vacancies, and a reasonable physical model was proposed based on experimental and simulation results. It is difficult to mediate the contradiction between mobility and threshold voltage for the single channel. Via a heterojunction channel strategy, desirable TFT performances, with mobility of 12.5 cm^2^/Vs, threshold voltage of 1.2 V and Ion/Ioff of 3 × 10^9^, are achieved when the oxygen-vacancy-enriched layer gets close to the gate insulator (GI). The enhanced performances can be mainly attributed to the formation of two-dimensional electron gas (2DEG), the insensitive potential barrier and the reasonable distribution of oxygen vacancy. On the contrary, when the oxygen-vacancy-enriched layer stays away from GI, all the main performances degenerate due to the vulnerable potential well. The findings may facilitate the development and application of heterojunction channels for improving the performances of electronic devices.

## 1. Introduction

Amorphous oxide semiconductors (AOSs) are promising candidates for thin-film transistor (TFT) applications due to their outstanding comprehensive advantages, such as high mobility, low processing temperature and excellent scalability, compared with Si-based TFT devices [1,2,3]. Nowadays, AOSs can generally be divided into two types according to the key element of In, i.e., In-based and In-free ones. In-based AOSs usually exhibit better TFT performances, especially superior electron mobility, due to the significant orbital overlap in spherical s-orbitals, which dominate the electron transport and then the conduction of AOS materials [4,5]. However, the In element belongs to a rare and strategic resource that is expensive and toxic, which restricts its wide application, conversely [6]. Therefore, the development of novel In-free AOSs that are low cost, high performance and environmentally friendly is of great importance for future display applications. 

ZnSnO (ZTO) is one of the many potential In-free AOS materials due to the low cost and non-toxicity, and it has garnered high expectations in the next-generation display backplane technique [7], while the ordinary performances, especially the mobility, remain to be further enhanced to compete with In-based AOSs. Although the device performances can be enhanced through material modification, it is still difficult to achieve a good comprehensive performance because the improvement in an index is usually accompanied by the deterioration of others [8,9,10]. In addition, some advanced preparation techniques are also adopted to enhance ZTO TFT performances, such as atomic layer deposition (ALD) and special annealing process, whereas there are compatibility issues with mainstream mass-production processes [11,12]. TFT structure design, in particular the multilayer channel, is another key method to manipulate the device performance and has attracted much attention recently. However, the intrinsic mechanism is still controversial, and the improvement is limited due to some non-ideal effects [13,14,15].

Compared with the heterojunction channel equipped with two different materials, the homojunction channel may facilitate the electron transport due to the superior interface quality and reduced interface scattering resulting from a continuous material structure [16]. In this work, the high performance of ZTO TFT, comparable to prevailing InGaZnO (IGZO) TFT, is achieved by using a homojunction strategy to manipulate the distribution of oxygen vacancies in the active layer channel. The formation of two-dimensional electron gas (2DEG) in the homojunction interface is the main reason for the enhanced TFT performances. Finally, the possible physical model and band structure of the homojunction ZTO TFT are proposed via experimental and simulation investigations. 

## 2. Materials and Methods

**Figure 1:**
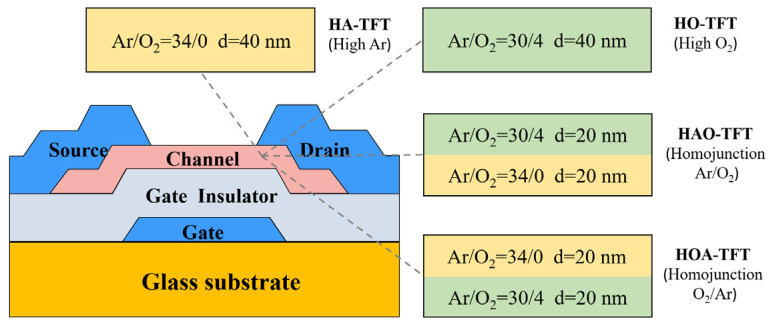
A schematic diagram of ZTO TFT with single/homojunction channel.

Bottom structural ZTO TFTs were fabricated on a glass substrate via magnetron sputtering with radio frequency (RF) power. ZTO thin film was deposited from ZTO ceramic target with Zn: Sn = 2: 1, and the detailed deposition parameters are as follows. The RF power, base pressure, working atmosphere (Ar/O_2_) and pressure are 140 W, 5 × 10^−4^ Pa, 32 sccm/2 sccm and 0.5 Pa, respectively. Firstly, 100 nm Mo thin film was deposited and patterned to form gate electrodes. Then, 200 nm SiO_2_ was deposited as gate insulator (GI) via plasma-enhanced chemical vapor deposition (PECVD). Afterwards, about 40 nm ZTO thin films were sputtered with different Ar/O_2_ ratios to regulate the distribution of oxygen vacancies; 20 nm ZTO active layer was then deposited in high-Ar condition (Ar/O_2_ = 34/0) and high-O_2_ condition (Ar/O_2_ = 30/4) to form homojunction channel configuration. In addition, 40 nm ZTO single channels with high-Ar and high-O_2_ atmosphere were also separately prepared for contrast. Subsequently, the ZTO thin films were annealed at 350 °C for 1.5 h to form a dense structure before patterning. More experimental details can be seen in our previous work [17]. As shown in Figure 1, the TFT devices with four different channels were abbreviated as HA-TFT, HO-TFT, HAO-TFT and HOA-TFT, respectively. Finally, 100 nm Mo thin film was sputtered and patterned to form source/drain electrodes. The electrical performances were measured using a semiconductor parameter analyzer (B1500A, Agilent, Santa Clara, CA, USA) in air and dark conditions.

## 3. Results and Discussion

**Figure 2:**
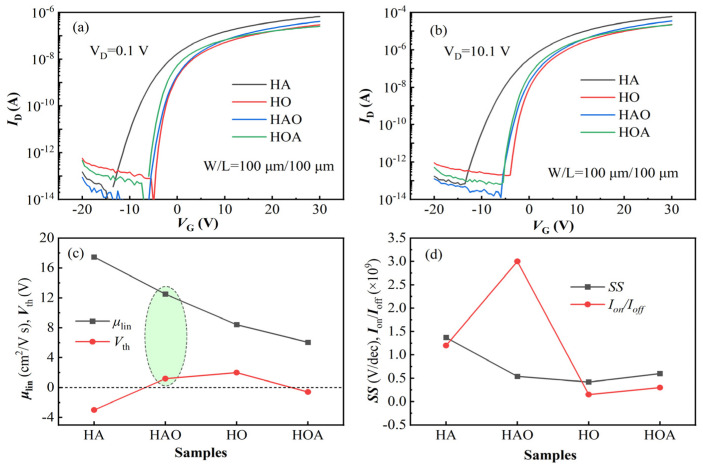
Transfer characteristic curves of the ZTO TFTs at (**a**) *V*_D_ = 0.1 V, (**b**) *V*_D_ = 10.1 V. (**c**,**d**) The extracted electrical parameters.

Figure 2a,b show the transfer characteristic curves of the ZTO TFT with different channel configurations and different drain voltages (*V*_D_). Generally, all the ZTO TFTs exhibit normal switch characteristics and ultra-low off-state current, ~10^−12^ A level. In addition, the ZTO TFT prepared via the channel in high-argon conditions (HA-TFT) shows a noticeable negative shift and an increased on-state current compared with others, which can be attributed to the increased electron concentration resulting from the ionized oxygen vacancies. For further comparison, the detailed electrical parameters were extracted from I-V curves according to the following equations [18,19].

(1) $$I_{D}^{}=\frac{W}{L}\mu_{\mathrm{lin}}C_{OX}[(V_{G}-V_{th})V_{D}-\frac{V_{D}^{2}}{2}]$$

(2) $$\mathrm{SS}=\frac{dV_{G}}{d(\log I_{D})}$$

(3) $$V_{\mathrm{th}}=V_{G};@(I_{D}=100\;nA\times \frac{L}{W},\;V_{D}=10.1\;V)$$

where *I*_D_, *V*_G_, *C*_ox_, *μ*_lin_, *SS*, *V*_th_, *W* and *L* are drain current, gate voltage, areal capacitance of gate dielectric, liner mobility, subthreshold swing, threshold voltage, width and length of the active layer, respectively. Here, the *V*_th_ is defined by the constant current method according to Equation (3). That is, the *V*_th_ was extracted as the *V*_G_ corresponding to an *L*/*W*-normalized *I*_D_ of 100 nA when *V*_D_ = 10.1 V. All the extracted electrical parameters are summarized in Table 1 and plotted in Figure 2c,d.

As shown in Figure 2c, the HA-TFT shows high electron mobility, while *V*_th_ is negative due to the excessive content of background electrons. On the contrary, the electron mobility of ZTO TFT prepared via the channel in high-oxygen conditions (HO-TFT) is low, and the *V*_th_ is positive due to the finite electrons ionized from oxygen vacancy. For the homojunction channels, when the oxygen-vacancy-enriched ZTO layer is close to the GI (HAO-TFT), high mobility and desirable threshold voltage were achieved. In the opposite case (HOA-TFT), not only the mobility declines but also the *V*_th_ becomes negative. The variations in *V*_th_ in the homojunction channels can be explained as follows. The back channel with fewer oxygen vacancies is favored for achieving positive *V*_th_ due to the high insulation, which hardly needs more gate voltage to deplete the conduction electrons. On the contrary, when the back channel is conductive, negative voltage is essential to deplete excessive electrons to achieve an acceptable off-state current. Figure 2d shows the variations in *SS* and current ratio between the on-state and off-state (*I*_on_/*I*_off_). The *SS* of HA-TFT is obviously higher than others, which can be plausibly attributed to the poor interface quality due to the considerable content of trap-state oxygen vacancy [20]. In addition, all the *I*_on_/*I*_off_ is above 10^8^, and the highest *I*_on_/*I*_off_ (3 × 10^9^) is obtained in HAO-TFT.

**Figure 3:**
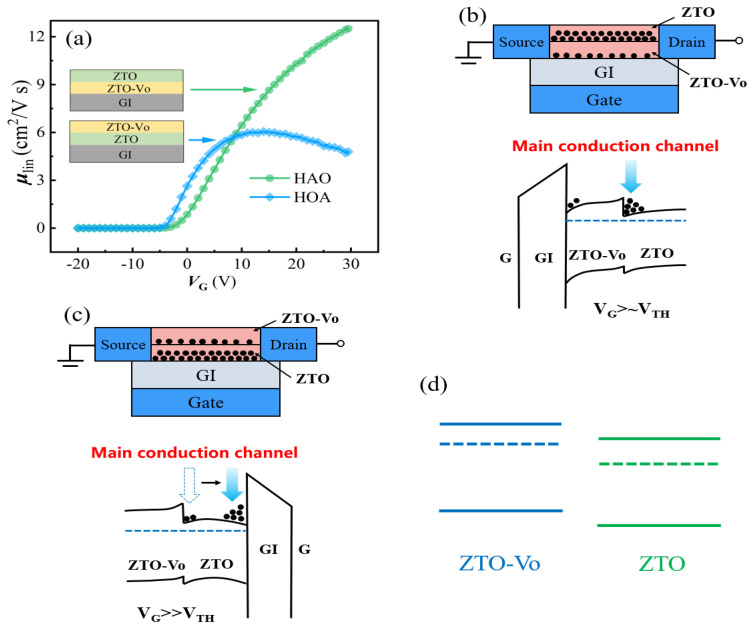
(**a**) The extracted linear mobility of ZTO TFT with respect to V_G_. (**b**,**c**) The working model and the energy band structure of homojunction. (**d**) The proposed energy band structure of ZTO materials with and without oxygen vacancy.

It is necessary and interesting to investigate the intrinsic conduction mechanism for the homogeneous junction channel. Generally, the mechanisms related to multilayer channels include charge compensation, composite channel and a 2DEG channel. To possibly distinguish the dominant mechanism, the linear mobilities with respect to different gate voltages are extracted, as shown in Figure 3a. With an increase in *V*_G_, the μlin of HAO-TFT increases gradually, while that of HOA-TFT increases first and then declines. Hence, it is believed that the electron conduction mechanism is different across the two devices. Figure 3b,c show the possible electron transport models and energy band structures. The electrons transfer from one layer to another due to the difference in electron concentration and then reach an equilibrium state under the effect of the built-in electric field, not only resulting in the alignment of Fermi energy levels but also the bending of the conduction band (CB) and valence band (VB). In the case of HAO-TFT, electrons are localized in the interface between the ZTO and oxygen-vacancy-enriched ZTO (ZTO-Vo) active layer, and a quantum well may be formed on the ZTO side and serve as the main conduction channel. It is hardly affected by the gate voltage, even at a considerable value, and accounts for the continuous increase in mobility with respect to *V*_G_. Electron transport in quantum wells is much easier due to the reduction in scattering and electron trap [21]. In addition, according to the percolation conduction theory, the mobility is, overall, positively correlated to the electron concentration due to the relatively decreased electron conduction barriers [22], which is opposite to the case of silicon-based semiconductor materials. That is, the enhancement in mobility also results from the accumulation of electrons in the quantum well. In the case of HOA-TFT, the quantum well is susceptible to an external electric field, which would be opened when the *V*_G_ reaches a certain level, resulting in a shift in the main conduction channel from the homojunction interface to the front interface [23], as illustrated in Figure 3c. The interface quality of the front channel is generally lower than that of the homojunction interface due to the obvious differences in material structures between SiO_2_ and ZTO, which could result in significant lattice mismatch and more dangling bonds. Then, electron transport is restricted, and the decline in mobility occurs when the *V*_G_ is high. Based on the above analyses, the basic energy band structure of ZTO with and without oxygen vacancy can be inferred, as shown in Figure 3d. That is, the formation of an oxygen vacancy would elevate the energy of both CB and the Fermi level. Simultaneously, the energy difference between CB and the Fermi level is reduced and results in the increased content of electrons, which is consistent with the reported literature [24,25]. 

**Figure 4:**
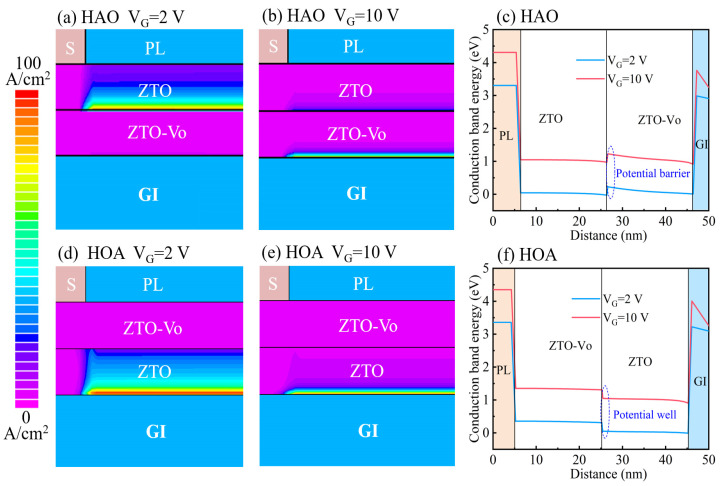
The simulated current density and conduction band structure. (**a**–**c**) and (**d**–**f**) are the cases for HAO-TFT and HOA-TFT, respectively.

To further confirm the rationality of the proposed physical model and electronic transport behaviors, TFT device simulations based on Silvaco TCAD were performed using the ATLAS module, as shown in Figure 4. The simulated current density of HAO-TFT is depicted in Figure 4a,b. When the applied gate voltage is not too high (*V*_G_ = 2 V), the main conduction channel is located at the homojunction interface between the ZTO layer and ZTO-Vo layer. More specifically, the electrons transport in the ZTO layer, while the conduction channel partially shifts to the front interface between ZTO-Vo and GI when the *V*_G_ increases to 10 V. It indicates that the electrons would preferentially transport at the homojunction interface, unless the *V*_G_ is high enough. This behavior can be attributed to the formation of potential barriers on the side of the ZTO-Vo layer, evident from the simulated conduction band structure (Figure 4c). An upward step-like potential barrier was formed, and more energy is needed for electrons to overcome the energy barrier for conduction channel migration. In the case of HOA-TFT (Figure 4d,e), the conduction channel already exhibited a movement tendency to the front interface between ZTO and GI, even at a low *V*_G_, and the increase in *V*_G_ exacerbates the migration behavior of the main conduction channel because the downward step-like energy potential well formed on the side of the ZTO layer can be more easily opened by the gate bias voltage due to the shallow depth, as shown in Figure 4f. That is, the stabilities of the potential barrier and potential well on gate voltage are different, and the order of homojunction/heterojunction layers is critically important to the TFT performance. It is also worth noting that the mobility of HAO-TFT would decline as well when the main conduction channel transfers to the front interface at a high enough gate voltage. The key advantage of HAO-TFT is that the gate voltage ranges of continuously increased mobility are greatly extended, compared with HOA-TFT, which is significant for practical applications. All the simulated results are consistent with the experiment results and the proposed physical models. This work demonstrates the feasibility of an enhancement in TFT performance by adopting a homojunction channel strategy, which is generalizable to other semiconductor materials and electronic devices.

## 4. Conclusions

In summary, the high performance of ZTO TFTs was achieved via a homojunction channel strategy by elaborately manipulating the distribution of oxygen vacancies, and the resulting TFT performance was systematically investigated through experimental and simulation methods. Simple oxygen defect regulation is insufficient to achieve excellent comprehensive performance. Although the mobility can be boosted by elevating the dose of oxygen vacancy, it is accompanied by a significantly negative threshold voltage. On the contrary, the suppressed oxygen vacancy leads to a decrease in mobility and an increase in threshold voltage. By using a heterojunction channel strategy, the contradiction between mobility and threshold voltage was alleviated. High TFT performances were achieved in HAO-TFT, and the optimized mobility, threshold voltage and *I*_on_/*I*_off_ were 12.5 cm^2^/Vs, 1.2 V and 3 × 10^9^, respectively, which can be attributed to the synergistic effects of the 2DEG channel, insensitive potential barrier and reasonable configuration of sub-channel layers. As a comparison, both the mobility and the threshold voltage of HOA-TFT are inferior to those of HAO-TFT, due to the susceptible potential well. All of this indicates that the homojunction strategy is effective in manipulating the electron transport behavior and, thus, the performances of electronic devices.

## Figures and Tables

**Table 1:** Summary of electrical parameters of ZTO TFT.

Devices	*μ*_lin_ (cm^2^/V s)	*V*_th_ (V)	*SS* (V/dec.)	*I*_on_/*I*_off_
HA-TFT	17.46	−3.0	1.37	1.2 × 10^9^
HAO-TFT	12.51	1.2	0.54	3.0 × 10^9^
HO-TFT	8.42	2.0	0.42	1.5 × 10^8^
HOA-TFT	6.05	−0.6	0.60	3.0 × 10^8^

## Data Availability

The data are available upon reasonable request from the corresponding author.
